# Development of Vaginal Pseudoaneurysm 3 Years after Cesarean Section Possibly Induced by Anticoagulant and Antiplatelet Therapies

**DOI:** 10.1155/2020/6196575

**Published:** 2020-03-24

**Authors:** Yui Kinjo, Tadatsugu Kinjo, Keiko Mekaru, Hayase Nitta, Hitoshi Masamoto, Yoichi Aoki

**Affiliations:** Department of Obstetrics and Gynecology, Graduate School of Medicine, University of the Ryukyus, 207 Uehara Nishihara, Okinawa 903-0215, Japan

## Abstract

Pseudoaneurysms generally develop when an arterial puncture site is inadequately sealed. We encountered a case of vaginal pseudoaneurysm that developed 3 years after cesarean section in a 35-year-old gravida 7 para 4 woman who was prescribed with anticoagulant and antiplatelet drugs after surgeries for ventricular septal defect and aortic valve replacement. Pelvic computed tomography scan revealed a large mass, which showed a dappled contrast filling on the arterial phase, located in the posterior vaginal wall. The vaginal pseudoaneurysm was completely occluded by embolization of the left vaginal artery. Anticoagulation and antiplatelet therapies can be potential causes of spontaneous pseudoaneurysm rupture. Extrauterine pseudoaneurysm has a long period of time between cesarean section and pseudoaneurysm discovery. Considering that pseudoaneurysm shows different clinical features for each patient, we should always consider pseudoaneurysm when we assess a patient with postpartum hemorrhage.

## 1. Introduction

Pseudoaneurysms (PAs) generally develop when an arterial puncture site is inadequately sealed. Uterine PA mainly shows postpartum vaginal bleeding after cesarean section (CS). Considering vascular wall vulnerability due to changes in hormonal and circulatory dynamics during pregnancy, PA can occur after not only invasive procedures, such as CS and dilatation and curettage (D&C), but also a normal vaginal delivery [1]. Typical cases of uterine PA exhibit vaginal bleeding 2 weeks to 1 month after CS [2, 3].

We encountered a case of vaginal PA that developed 3 years after CS in a patient who had been prescribed with anticoagulant and antiplatelet drugs until the PA rupture.

## 2. Case Report

**Figure 1:**
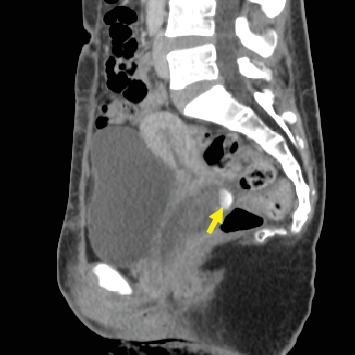
Pelvic computed tomography scan (sagittal view) reveals a large mass, which shows a dappled contrast filling on the arterial phase, located in the posterior vaginal wall (arrow).

**Figure 2:**
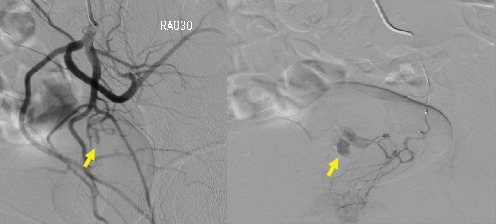
Pelvic angiography shows pseudoaneurysm formation in the left vaginal artery (arrows). (a) Angiography for the left internal iliac artery. (b) Selective angiography for the left vaginal artery.

**Figure 3:**
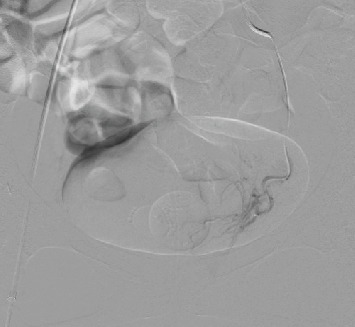
Complete occlusion of the left vaginal pseudoaneurysm by embolization of the left vaginal artery.

A 35-year-old gravida 7 para 4 woman had histories of CS for four times and D and C for three times. The last CS was performed 3 years ago. Furthermore, surgeries for ventricular septal defect and aortic valve replacement were performed when she was 14 and 24 years old, respectively. Thereafter, anticoagulant and antiplatelet drugs (warfarin potassium at 2 mg/day and clopidogrel sulfate at 75 mg/day, respectively) had been prescribed until the PA rupture. Eventually, she complained of atypical vaginal bleeding for several days and then visited our department. Ultrasonography showed no abnormal findings in the uterus at that time. However, 3 days later, she experienced profuse vaginal bleeding and thereby was admitted to our hospital. Her blood pressure and pulse rate were 100/70 mmHg and 106 beats/min, respectively. Her laboratory results were as follows: white blood cell count, 3.7 × 10^3^/*μ*l; hemoglobin count, 6.2 g/dl; platelet level, 210 × 10^3^/*μ*l; and prothrombin time-international normalized ratio, 1.95. However, second ultrasonography could not detect any uterine abnormality; therefore, contrast-enhanced computed tomography (CT) scan was performed. Pelvic CT scan revealed a large mass, which showed a dappled contrast filling on the arterial phase, located in the posterior vaginal wall (Figure 1). Thereafter, vascular radiologists performed emergency pelvic angiography, which revealed PA formation in the left vaginal artery (Figure 2). The PA was completely occluded by embolization of the left vaginal artery (Figure 3). Embolization procedures were performed with metallic coils and a gelatin sponge (Gelpart®; Nippon Kayaku, Tokyo, Japan), which measured 2 mm in diameter, via a microcatheter (Renegade® infusion catheter (2.5 Fr); Boston Scientific, Tokyo, Japan). Postembolization course was uneventful.

## 3. Discussion

A potential cause of spontaneous PA rupture in our case may be the use of anticoagulation and antiplatelet therapies after aortic valve replacement. Our patient had been prescribed with anticoagulant and antiplatelet drugs until the PA rupture. Several reports pointed out anticoagulation therapy as the possible cause of ruptured PA [4, 5]. According to a prospective study, failure of thrombosis was associated with considerably large PAs and the concomitant use of anticoagulation or antiplatelet agents [6]. Furthermore, late postpartum hemorrhage due to ruptured PA also occurs in patients with von Willebrand disease [7, 8]. In general, PAs are thrombosed spontaneously. Therefore, PA is formed as a result of repeated bleeding and thrombus due to abnormal hemostasis [4–8]. Hence, an underlying bleeding disorder or anticoagulation therapy should be considered in patients presenting with PA rupture-induced postpartum hemorrhage.

PA can occur after 3 years of CS. Toursarkissian et al. reported that spontaneous thrombosis of PA occurred in 88% of cases with small PAs at a mean of 23 days [9]. Isono et al. reviewed that the average time between preceding CS and PA is 14.5 days [2], whereas Baba et al. reported that it is at a median of 28 days and a maximum of 140 days [10]. Accordingly, a period of 3 years for PA to occur, as revealed in our case, is extremely rare. However, a large internal iliac pelvic PA combined with arteriovenous fistula appeared in a patient 20 years following an elective CS [11]; in another case, the time period between the CS and PA occurrence was approximately 10 years with no other potential causative factors [12]. Interestingly, Matsubara et al. speculated that extrauterine PA has a long period of time between CS and PA discovery [1].

The PA in our case developed from the vaginal artery and was located in the posterior vaginal wall, not in the uterus. Dohan et al. reported that seven out of 18 cases with PA after CS originated from the vaginal artery [3]. PA appearing in the internal iliac artery [11] or the Douglas pouch [12] and PA showing a pelvic mass [13] were also reported. Intrauterine PA undergoes morphological and functional dynamic changes in the uterus after CS, and it is easily damaged and ruptured. However, extrauterine PA does not suffer from such dynamic changes and is difficult to rupture; thus, time lag occurs between CS and extrauterine PA discovery [1].

In conclusion, anticoagulation and antiplatelet therapies can be potential causes of spontaneous PA rupture. Extrauterine PA has a long period of time between CS and PA discovery. Considering that PA shows different clinical features for each patient, we should always consider PA when we assess a patient with postpartum hemorrhage.
